# Classification of Septic Shock Phenotypes Based on the Presence of Hypotension and Hyperlactatemia in Cats

**DOI:** 10.3389/fvets.2021.692528

**Published:** 2021-09-14

**Authors:** Roberta Troia, Francesca Buzzurra, Elena Ciuffoli, Giulia Mascalzoni, Armando Foglia, Ilaria Magagnoli, Francesco Dondi, Massimo Giunti

**Affiliations:** Department of Veterinary Medical Sciences, Alma Mater Studiorum- University of Bologna, Bologna, Italy

**Keywords:** cryptic, dysoxic, feline, vasoplegic, multi-organ dysfunction syndrome

## Abstract

**Background:** Three different phenotypes of septic shock based on changes in blood pressure and lactate are recognized in people. Dysoxic shock, representing the combination of fluid-refractory hypotension and hyperlactatemia, is characterized by greater disease severity and mortality compared to cryptic shock (hyperlactatemia alone) and vasoplegic shock (hypotension with normal blood lactate). Little is known about septic shock and specifically its phenotypes in cats.

**Objective:** To analyze the characteristics and prognostic implications of three septic shock phenotypes in cats with sepsis.

**Methods:** Cats with septic shock were prospectively included. Septic shock was defined by the presence of hypotension (mean blood pressure <60 mmHg) requiring vasopressor support and/or persistent hyperlactatemia (>4 mmol/L) and classified in three subgroups: *dysoxic shock, vasoplegic shock* and *cryptic shock*. Clinical and clinicopathological variables including APPLE_fast_ and APPLE_full_ scores, occurrence of multi-organ dysfunction syndrome (MODS; presence of at least two dysfunctional organs simultaneously) and outcome were compared among subgroups. Cats with sepsis showing normal blood pressure and lactate concentrations hospitalized during the study period were included as *uncomplicated sepsis*, and compared to cats with septic shock for selected variables. Length of hospital stay and mortality were evaluated in the whole study population. Odds ratios for mortality were calculated using logistic regression analysis. Significance was set at *P* < 0.05.

**Results:** The study enrolled 48 cats with uncomplicated sepsis and 37 cats with septic shock (dysoxic shock *n* = 17; vasoplegic shock *n* = 11; cryptic shock *n* = 7). Cats with dysoxic shock had significantly higher APPLE_fast_ and APPLE_full_ scores compared to vasoplegic and cryptic shock. Mortality rates were not significantly different among cryptic (57%), dysoxic (65%) and vasoplegic shock (91%), while MODS occurrence was significantly lower in cats with cryptic shock (57%) compared to patients affected by dysoxic (94%) and vasoplegic (100%) shock. Cats with septic shock had higher frequency of MODS and greater mortality rate than cats with uncomplicated sepsis.

**Conclusion:** Despite similar in-hospital mortality, cats with dysoxic and vasoplegic shock are characterized by having higher occurrence of multi- organ dysfunction compared to cats affected by cryptic shock. Results from this study suggest novel means of identifying high-risk subgroups of septic cats.

## Introduction

Sepsis, the life-threatening organ dysfunction caused by a dysregulated host response to infection, is a global health burden affecting critically-ill people and veterinary patients (1–3). According to the Third International Consensus Definition Sepsis-3 septic shock is defined as a subset of sepsis in which particularly profound circulatory, cellular, and metabolic abnormalities are associated with a greater risk of mortality than with sepsis alone. Risk-adjusted in-hospital mortality is significantly higher in human patients with fluid-refractory hypotension (systolic blood pressure [SBP] <90 mmHg or mean blood pressure [MAP] <60 mmHg) and hyperlactatemia (blood lactate concentrations >2 mmol/L) compared with either hyperlactatemia alone or with fluid resistant hypotension requiring vasopressors but with normal blood lactate concentrations (1).

A reclassification of the spectrum of septic patients using lactate had been proposed in people recognizing three different phenotypes of shock: dysoxic, vasoplegic, and cryptic (4–6). *Dysoxic shock*, defined by the combination of hyperlactatemia and hypotension requiring vasopressor support, is characterized by higher illness severity scores, higher mean number of organ dysfunctions and lower survival rates (4–6). *Vasoplegic shock* is a more favorable phenotype where patients requiring prolonged vasopressor therapy for hypotension do not develop hyperlactatemia (7). Septic humans with vasoplegic shock usually present lower severity scores and lower rates of hospital mortality, suggesting a more preserved global homeostasis despite the evidence of circulatory stress (7–9). Finally, the presence of occult hypoperfusion characterized by persistent hyperlactatemia in absence of hypotension is defined as *cryptic shock*. Severity of illness and mortality in patients with cryptic shock could be equivalent to those in patients with vasoplegic shock. This suggests that sole standing hyperlactatemia increases risk of death independently from vasopressor need. Therefore, such patients might require the same level of intensive care as hypotensive patients (4–6, 10, 11). Stratification of septic patients based on these different septic shock phenotypes could be of fundamental epidemiological and prognostic importance. Moreover, hyperlactatemia may be considered a predictor of mortality, regardless of the phenotypes of septic shock (4–6, 9–11).

In small animals, septic shock is defined as a state of fluid-refractory hypotension requiring vasopressor support and is reportedly associated with higher mortality rates compared to uncomplicated sepsis (3, 12–15). However, feline septic shock is not commonly investigated (3, 15), and the stratification and the prognostic impact of the different septic shock phenotypes based on hyperlactatemia and fluid-refractory hypotension have not yet been documented.

The aims of the current study were: (1) to analyze the prevalence and characteristics of different phenotypes of septic shock, defined by the presence of fluid-refractory hypotension requiring vasopressor support and/or hyperlactatemia, in a population of cats with septic shock hospitalized in an intensive care unit (ICU); (2) to compare the clinical presentation and outcome of this population of cats with septic shock to a cohort of cats with uncomplicated sepsis. We hypothesized that cats with septic shock have greater illness severity and experience worse outcomes compared to cats with uncomplicated sepsis, and that the feline septic shock phenotypes resemble the ones identified in humans in terms of prevalence, risk for organ dysfunction and mortality.

## Materials and Methods

### Study Population and Setting

Cats with sepsis presented to a Veterinary University Hospital between April 2018 and November 2020 and hospitalized in the ICU for at least 12 h were eligible for inclusion. Cats were diagnosed with sepsis based on the presence of infection confirmed by means of cytology, microbiology, histopathology or real-time polymerase chain reaction, plus at least one of the following: fulfill two or more Systemic Inflammatory Response Syndrome (SIRS) criteria (2); a serum amyloid-A (SAA) concentration above the laboratory reference interval (RI) (>10 μg/mL); evidence of at least one new-onset organ dysfunction. Cats with sepsis were diagnosed with septic shock if they had persistent hyperlactatemia (lactate >4 mmol/L for >12 h despite fluid resuscitation or clinical euvolemia) and/or hypotension (MAP <60 mmHg) requiring vasopressor support despite adequate fluid resuscitation (1). Cats not fulfilling septic shock criteria were placed in the uncomplicated sepsis category for statistical analysis. Serial monitoring of blood lactate was regularly performed at the end of fluid resuscitation, and then at different time intervals at the clinician discretion. Cats diagnosed with neoplasia, as well as cats discharged against medical advice or euthanized for financial reasons were excluded. The study was approved by the local Institutional Animal Care and Use Committee (protocol number ID 846).

### Data Collection

Signalment, body weight, body condition score (BCS; where 1–3/9 was defined as underweight condition, 4–6/9 as ideal and 7–9/9 as obese), medical history including comorbidities, previous and current treatments, physical examination including non-invasive systolic blood pressure measurement (SunTech® Vet20^TM^ Veterinary Blood Pressure Monitor, SunTech Medical, Inc., USA) were recorded at study enrollment. Attending ICU clinicians were responsible for the clinical management of the patients. Blood was collected by venipuncture with vacuum system according to standard operating procedures. Blood gas, electrolytes, and blood lactate measurements were performed using point-of-care analyzers (ABL800 FLEX, Radiometer Medical ApS, Denmark). Complete blood count and serum chemistry analyses including SAA evaluation were performed using automated analyzers (ADVIA 2120, Siemens Healthcare Diagnostics, Tarrytown, NY; Olympus AU 480, Olympus/Beckman Coulter, Brea, CA). SAA concentrations were measured on serum samples using a commercial immunoturbidimetric assay designed for human SAA (LZ Test Eiken SAA, Eiken Chemical, Tokyo, Japan), as previously validated for cats and applied in our lab (16, 17). The 5-variables feline Acute Patient Physiologic and Laboratory Evaluation fast (APPLE_fast_) score was calculated in cats with uncomplicated sepsis and septic shock (18). Multiorgan dysfunction syndrome (MODS) was defined as the presence of at least two dysfunctional organs simultaneously, as previously reported (3) and as depicted in Table 1. Outcome was defined as survival to hospital discharge, death or euthanasia due to grave prognosis.

**Table 1:** Criteria for organ dysfunction in cats (3).

**Organ dysfunction**	**Criteria**
Respiratory dysfunction	Sp*O*_2_ <95% in room air, need of oxygen therapy or mechanical ventilation
Hepatic dysfunction	Bilirubin > 0.7 mg/dL (11.97 μmol/L) in absence of hemolysis or biliary obstruction
Renal dysfunction	Serum creatinine > 1.8 mg/dL (159.16 μmol/L) and/or increase of ≥ 0.3 mg/dL (26.5 μmol/L) from baseline and/or oliguria (urine output <1 ml/kg/h over 6 h)
Cardiovascular dysfunction	SBP <90 mmHg or MAP <60 mmHg in euvolemic patients requiring vasopressors
Hemostatic dysfunction	PT > 15 s and/or aPTT > 20 s and/or platelet count <100,000/m*m*^3^

*aPTT, activated partial thromboplastin time; MAP, mean arterial pressure; PT, prothrombin time; SBP, systolic blood pressure. Cut-off values for selected clinicopathological variables (serum creatinine, bilirubin, PT, aPTT) were based on the upper bound of the respective reference intervals of our clinical pathology laboratory*.

### Additional Data Collection in Cats With Septic Shock

Additional data were systematically recorded in cats diagnosed with septic shock: the 10-variables APPLE full score (APPLE_full_); intravenous fluid volumes administered during resuscitation and during the first 24 h of hospitalization; type and duration of vasopressor support.

### Septic Shock Phenotypes

Cats with septic shock were divided into subgroups according to three different phenotypes, as previously reported in humans: (1) cryptic shock, defined as hyperlactatemia (>4 mmol/L) in the absence of vasopressor support; (2) vasoplegic shock, defined as hypotension (MAP <60 mmHg) requiring vasopressor support in the absence of hyperlactatemia; (3) dysoxic shock, defined by the combination of hypotension requiring vasopressor support and hyperlactatemia (4–6, 10).

### Statistical Analysis

Normality of data distribution was assessed graphically and using the D'Agostino Pearson test. Data were reported as median and range (minimum-maximum value), or mean ± standard deviation (SD), based on their distribution. The Mann-Whitney U-test, Student's *t*-test and the Kruskal-Wallis test with *post-hoc* comparison were used to compare continuous variables among groups (uncomplicated sepsis vs. septic shock; cryptic vs. vasoplegic vs. dysoxic shock; cryptic shock vs. uncomplicated sepsis; survivors vs. non-survivors). Categorical variables were compared among groups using the Fisher's exact test and the Chi squared test. Associations between variables of interest and death for the overall population of septic cats were examined by univariate regression analysis, and the variables that were associated with the outcome (*P* < 0.05) were included in a multivariable regression model (stepwise selection); results were presented as odds ratio (OR) and 95% confidence interval (CI). For all tests applied, a value of *P* < 0.05 was considered significant. Statistical analysis was performed using a statistical software package (MedCalc Statistical Software version 19.1.3 Ostend, Belgium 2019).

## Results

The study included 85 cats. From those 48/85 (56%) had uncomplicated sepsis and 37/85 (44%) had septic shock. Among the cats with uncomplicated sepsis, there were 12 spayed females, nine intact females, 19 neutered males and eight intact males. There were 39 domestic shorthair cats and nine purebred cats (three Siamese cats, two Persian cats, one Birman, one Ragdoll, one Bengal, one Main Coon). The median age was 4 years (0.16–17) and the mean body weight was 3.7 ± 1.6 kg. The BCS was between 1 and 3 in 24/48 (50%), between 4 and 6 in 21/48 (44%) cats, and 7 in 3/48 (6%) cats, respectively. Twenty-nine of 48 (60%) cats had at least two of four SIRS criteria. Underlying causes for sepsis included pyelonephritis (*n* = *15*), trauma with infected wounds (*n* = 7), pyothorax (*n* = *6*), feline panleukopenia virus infection (*n* = *6*), septic peritonitis (*n* = *5*), bacterial cholangitis (*n* = *4)*, pyometra (*n* = *4*), abdominal abscess (*n* = *1*).

Among the cats affected by septic shock, there were 12 spayed females, eight intact females, ten neutered males and seven intact males. There were 36 domestic shorthair cats and one Bengal. The median age was 6 years (0.16–16) and the mean body weight was 3.5 ± 1.8 kg. The BCS was between 1 and 3 in 20/37 (54%), between 4 and 6 in 13/37 (35%), and 7 in 4/37 (11%) cats, respectively. Twenty-eight of 37 (76%) cats met at least two of four SIRS criteria. Underlying causes for septic shock included trauma with infected wounds (*n* = 11), feline panleukopenia (*n* = *7*), bacteremia in the course of heterogeneous diseases (2 acute gastroenteritis, 1 diabetic ketoacidosis, 1 ingestion of caustics; *n* = *5*), pyelonephritis (*n* = *5*), septic peritonitis (*n* = *4*), bacterial cholangitis (*n* = *3)*, pyothorax (*n* = *2*), abdominal abscesses (*n* = *1*).

### Comparison Among Septic Shock Phenotypes

At the time of inclusion 17/37 (46%) cats were affected by dysoxic shock, 11/37 (30%) had vasoplegic shock, while 7/37 (19%) had cryptic shock. Two cats with persistent hypotension requiring vasopressor support were excluded from phenotyping because no contextual blood lactate measurement was performed.

**Figure 1:**
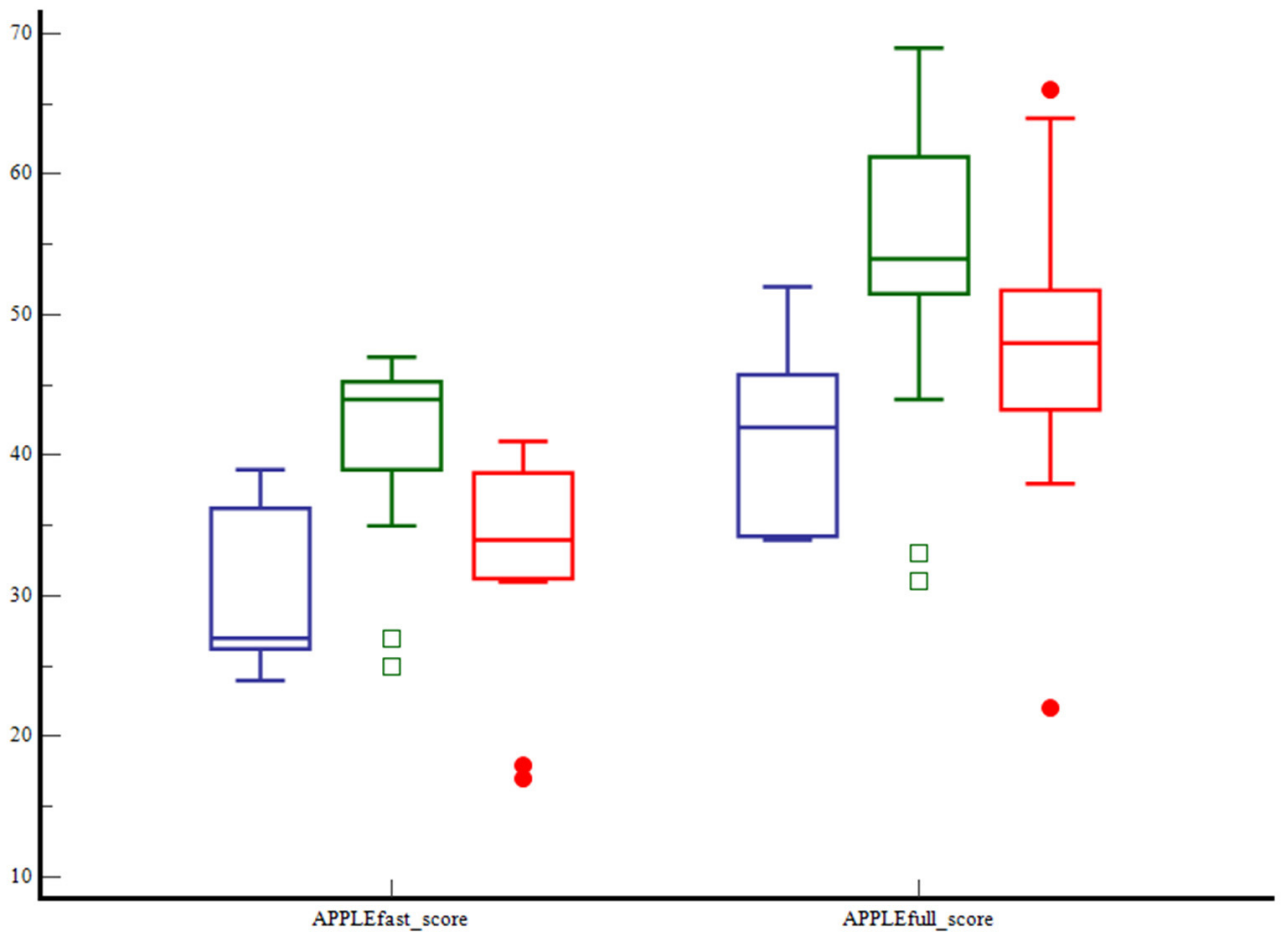
Box and whisker plots comparing the APPLE_fast_ and APPLE_full_ scores among cats with cryptic shock (*n* = 7, in blue), dysoxic shock (*n* = 17, in green), and vasoplegic shock (*n* = 11, in red). The central lines represent the median, the boundaries of the boxes represent the interquartile range and the whiskers represent the minimum and maximum values. Cats with dysoxic shock had significantly higher APPLE_fast_ and APPLE_full_ scores compared to cats with cryptic and vasoplegic shock (*P* < 0.001 and *P* < 0.015, respectively).

**Figure 2:**
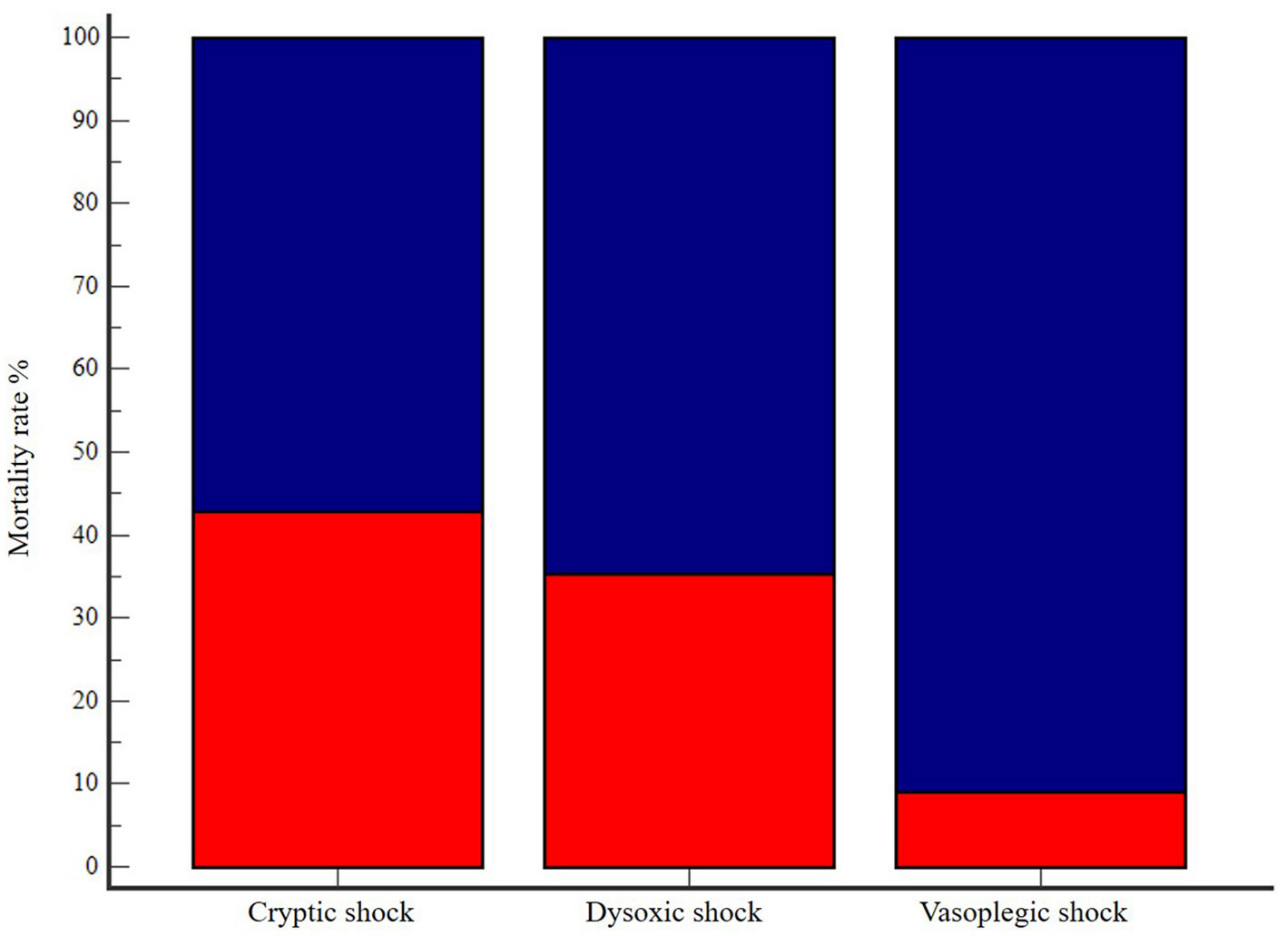
Bar chart with 100% stacked columns showing mortality rate (in dark blue) in cats with cryptic shock (*n* = 7, 57%), dysoxic shock (*n* = 17, 65%), and vasoplegic shock (*n* = 11, 91%). The difference was not statistically significant (*P* = 0.09).

**Figure 3:**
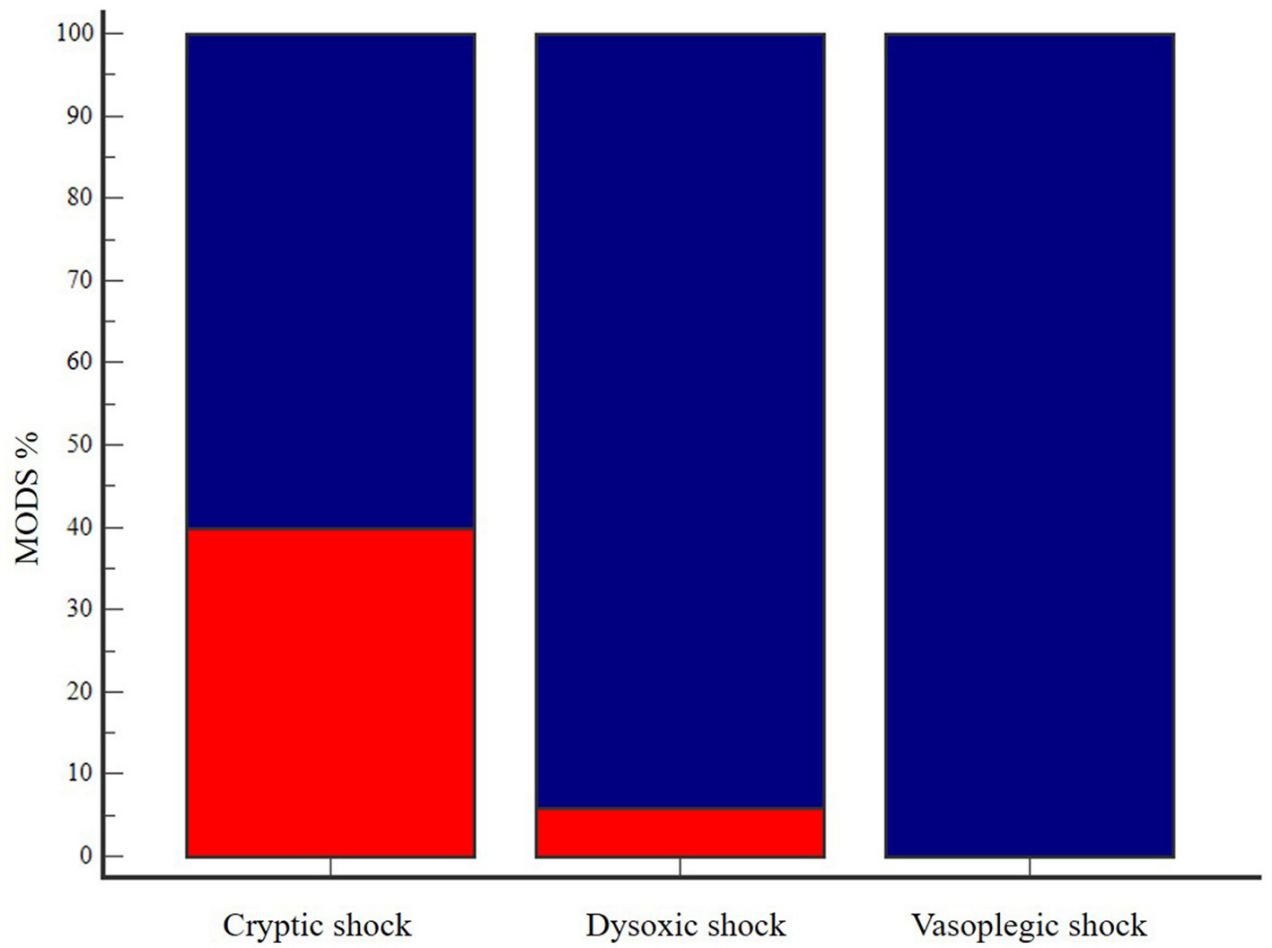
Bar chart with 100% stacked columns showing the percentage in MODS occurrence (in dark blue) in cats with cryptic shock (*n* = 7, 57%), dysoxic shock (*n* = 17, 94%) and vasoplegic shock (*n* = 11, 100%). The difference was statistically significant (*P* = 0.02). MODS, multiorgan dysfunction syndrome.

Clinical and clinicopathological characteristics of the three septic shock phenotypes are summarized in Table 2. Age, body weight and BCS were not different among groups. Cats with dysoxic shock had significantly higher median APPLE_fast_ and APPLE_full_ scores compared to cases from the other subgroups (Figure 1). The overall amount of intravenous fluids used for fluid resuscitation and during the first 24 h of hospitalization were not different in the three septic shock phenotypes. Length of hospital stay and mortality rates were not significantly different among cryptic, dysoxic and vasoplegic shock (Table 3, Figure 2). The frequency of MODS was significantly lower in cats with cryptic shock (57%, 4/7) compared to cases affected by dysoxic (94%, 16/17) and vasoplegic (100%, 11/11) shock (Figure 3).

**Table 2:** Clinical and clinicopathological variables results in cats with sepsis and septic shock.

**Variable**	**RI**	**Sepsis** **(*n =* 48)**	**Septic shock** **(*n =* 37)**	***P*-value**
**Clinical data**				
Body temperature (°C)	38–39	38.6 (32.0–40.6)	36.1 ± 2.3	**0.0001**
Heart rate (bpm)	160–220	180 ± 36	180 (100–230)	0.2652
Respiratory rate (rpm)	10–40	28 (20–100)	36 (20–100)	0.6345
SBP (mmHg)	120–170	120 ± 25	81 (51–180)	**<0.0001**
MAP (mmHg)	60–130	93 ± 21	62 ± 22	**<0.0001**
APPLE fast score		26 ± 7	36 ± 8	**<0.0001**
APPLE full score		/	49 ± 11	NA
SIRS criteria (*n*)		1.8 ± 1.0	2.3 ± 0.9	**0.0190**
Resuscitation fluid therapy (ml/kg)^*^		6.0 (5.0–50.0)	15.0 (0.5–37.0)	**0.0407**
Total fluid therapy/24 h (ml)		192.0 (11.0–860.0)	179.8 (48.0–811.0)	0.5856
Length of hospital stay		5 (0–28)	3 (0–25)	0.0827
**Blood gas analysis**				
pH	7.31–7.46	7.29 (6.94–7.42)	7.21 ± 0.14	0.3103
HC*O*_3_ (mmol/L)	18–22	17.1 ± 5.3	14.6 ± 4.8	**0.0356**
pC*O*_2_ (mmol/L)	32.7–44.7	38.4 (25.5–58.8)	36.3 ± 11.2	0.1270
BE (mmol/L)	−2.0–2.0	−8.9 ± 7.2	−12.1 ± 6.9	0.0649
Anion Gap (mmol/L)	12.0–16.0	16.6 ± 9.1	16.1 ± 7.6	0.7840
Ionized calcium (mmol/L)	1.10–1.41	1.22 ± 0.11	1.18 ± 0.19	0.0866
MetHb (%)	0.0–2.1	1.8 ± 1.1	1.7 (0.0–6.4)	0.5043
Lactate (mmol/L)^§^	0.5–2.0	2.1 ± 1.0	4.7 (0.6–26.0)	**<0.0001**
**Hematology**				
HCT (%)	24–45	30.0 ± 7.2	28.2 ± 9.0	0.2943
MCV (fL)	39.0–55.0	43.0 (36.4–54.4)	42.3 (35.8–56.9)	0.4422
MCHC (g%)	30–36	33.5 (29.1–42.1)	32.7 ± 1.7	0.0763
RDW (%)	14.1–18.4	14.9 (13.2–23.3)	15.7 (12.9–23.3)	0.1393
WBC (× 10^9^/L)	5.0–19.0	11.2 (0.4–66.0)	12.4 (0.0–96.5)	0.7312
Lymphocytes (× 10^9^/L)	1.5–7.0	1.1 ± 0.8	1.0 ± 0.8	0.2190
Neutrophils (× 10^9^/L)	2.0–12.5	8.8 (0.1–46.9)	7.3 (0.0–71.2)	0.7483
Monocytes (× 10^9^/L)	0.1–0.9	0.2 (0.0–3.3)	0.2 (0.0–1.5)	0.8352
Eosinophils (× 10^9^/L)	0.0–0.8	0.1 (0.0–0.7)	0.1 (0.0–23.1)	0.3801
Platelets (× 10^9^/L)	300.0–700.0	238.0 (46.0–472.0)	188.0 (7.0–534.0)	0.0834
MPV (fL)	10.0–15.5	17.1 ± 3.8	17.8 ± 4.2	0.4679
**Chemistry**				
Glucose (mg/dL)	75–160	141 (11–702)	135 (20–421)	0.5813
Creatinine (mg/dL)	0.80–1.80	1.17 (0.43–23.04)	1.78 (0.26–19.47)	0.7869
Urea (mg/dL)	15–60	61 (16–797)	102 (24–868)	0.1963
Total proteins (mg/dL)	6.0–8.0	7.0 ± 1.4	5.9 ± 1.4	**0.0010**
Albumin (mg/dL)	2.1–3.3	2.7 ± 0.6	2.3 ± 0.6	**0.0016**
A/G	0.5–1.2	0.7 ± 0.2	0.7 ± 0.2	0.6189
AST (U/L)	14–41	50 (17–1,459)	272 (18–2,099)	**0.0004**
ALT (U/L)	22–45	42 (4–1,285)	125 (4–5,268)	**0.0084**
GGT (mg/dL)	0.0–3.0	0.2 (0.1–2.8)	0.6 (0.1–49.8)	0.3001
ALP (U/L)	91–326	26 (2–89)	37 (1–312)	0.1736
CK (U/L)	0–120	321 (62–249,000)	920 (151–570,000)	**0.0007**
Total bilirubin (mg/dL)	0.00–0.35	0.26 (0.09–5.16)	0.82 (0.10–9.19)	**0.0008**
Phosphorus (mg/dL)	2.9–8.3	4.8 (2.2–31.6)	7.2 (2.3–33.7)	0.0702
SAA (mg/dL)	0–10	149 (1–338)	165 (1–338)	0.7216
**Coagulation**				
PT (sec)	9.0–15.0	8.6 (5.4–11.9)	10.3 (8.2–20.3)	**0.0006**
aPTT (sec)	9.0–20.0	17.5 (8.7–69.8)	30.0 (15.6–180.0)	**0.0020**

*Data are reported as mean ± standard deviation or median and range (minimum-maximum value), based on their distribution*.

*A/G, albumin to globulin ratio; ALP, alkaline phosphatase; ALT, alanine transaminase; APPLE, acute patient physiologic and laboratory evaluation; aPTT, activated partial thromboplastin time; AST, aspartate transaminase; BE, base excess; CK, creatine kinase; GGT, γ- glutamyltransferase; HCT, hematocrit value; MAP, mean blood pressure; MCHC, mean cell hemoglobin concentration; MCV, mean corpuscular volume; MetHb, methemoglobin; MPV, mean platelet volume; PT, prothrombin time; RDW, red cell distribution width; RI, reference interval; SAA, serum-amyloid A; SBP, systolic blood pressure; SIRS, systemic inflammatory response syndrome; WBC, white blood cell; NA, not applicable/not available. Bold indicates significance*.

* *Resuscitation fluid therapy (mL/kg) refers to the total amount of fluids given as bolus (either single or multiple) in case of hypovolemic shock*.

§ *Lactate concentration refers to the value measured at the time of study enrollment*.

**Table 3:** Clinical and clinicopathological variables in cats with dysoxic shock, cryptic shock, and vasoplegic shock.

**Variable**	**RI**	**Dysoxic shock (*n =* 17)**	**Cryptic shock (*n =* 7)**	**Vasoplegic shock (*n =* 11)**	***P*-value**
**Clinical data**					
Age (years)		6.0 (0.2–16.0)	9.0 (0.3–13.0)	8.0 (0.9–16.0)	NA
Weight (kg)		3.5 (0.8–9.4)	4.3 (1.0–5.7)	3.0 (2.0–7.1)	NA
APPLE fast score		44 (25–47) ^a^^,^^b^	27 (24–36) ^c^	34 (17–39) ^c^	**0.0007**
APPLE full score		54 (31–69) ^a^	42 (34–52) ^c^	48 (22–66)	**0.0158**
Resuscitation fluid therapy (ml/kg)		15.0 (5.0–37.0)	10.0 (2.5–15.0)	18.3 (2.8–35.0)	0.1821
Total fluid therapy / 24 h (ml)		216.0 (99.0–811.0)	137.0 (58.9–529.0)	153.2 (48.0–540.2)	0.3934
Noradrenaline (h)		16 (3–24)		15 (2–24)	**0.29**
Length of hospital stay		3 (0–25)	4 (0–16)	2 (0–7)	0.2397
**Hematology**					
HCT (%)	24.0–45.0	29.2 (9.9–47.5)	31.8 (18.8–38.8)	25.4 (10.9–38.2)	0.6813
WBC (× 10^9^/L)	5.0–19.0	15.2 (0.2–96.6)	6.5 (1.1–29.9)	18.0 (0.0–40.1)	0.3505
Lymphocytes (× 10^9^/L)	1.5–7.0	1.1 (0.0–2.5)	0.9 (0.3–1.5)	0.6 (0.0–2.8)	0.5889
Neutrophils (× 10^9^/L)	2.0–12.5	13.9 (0.0–71.2)	4.9 (0.0–27.6)	13.2 (0.0–35.9)	0.2735
Platelets (× 10^9^/L)	300.0–700.0	150.0 (7.0–534.0)	206.0 (9.0–511.0)	183.5 (20.0–242.0)	0.7394
**Chemistry**					
Creatinine (mg/dL)	0.80–1.80	1.85 (0.36–11.80)	0.93 (0.56–12.00)	1.90 (0.38–19.47)	0.3242
Total proteins (mg/dL)	6.0–8.0	5.1 (3.7–10.3)	5.9 (5.4–7.0)	6.3 (3.9–8.5)	0.3839
Albumin (mg/dL)	2.1–3.3	2.0 (1.4–3.5)	2.5 (1.8–3.1)	2.6 (1.4–3.5)	0.3901
AST (U/L)	14–41	650 (18–2,099)	227 (60–1,179)	130 (20–587)	0.1372
ALT (U/L)	22–45	552 (11–5,268) ^b^	56 (20–649)	72 (4–612) ^c^	**0.0380**
CK (U/L)	0–120	961 (208–570,000)	3,806 (151–84,805)	879 (162–67,167)	0.7764
Total bilirubin (mg/dL)	0.00–0.70	0.82 (0.10–7.46)	0.53 (0.16–7.09)	1.2 (0.36–9.19)	0.2528
SAA (mg/dL)	0–10	151 (1–281)	167 (26–242)	90 (6–338)	0.8628
***Coagulation***					
PT (sec)	9.0–15.0	11.4 (8.2–20.3)	9.4 (8.3–13.8)	10.1 (8.5–12.8)	0.3118
aPTT (sec)	9.0–20.0	28.0 (15.6–180.0)	23.6 (15.6–100.0)	34.9 (16.9–120.0)	0.4937

*Data are reported as median and range (minimum-maximum value)*.

*ALT, alanine transaminase; APPLE, acute patient physiologic and laboratory evaluation; aPTT, activated partial thromboplastin time; AST, aspartate transaminase; CK, creatine kinase; PT, prothrombin time; RI, reference interval; SAA, serum-amyloid A; WBC, white blood cell; NA, not applicable/not available*.

a *a significant difference from cryptic shock*;

b *a significant difference from vasoplegic shock*;

c *a significant difference from disoxic shock. Bold indicates significance*.

All cats with septic shock characterized by persistent fluid-refractory hypotension (dysoxic and vasoplegic shock) received norepinephrine as the first-line vasopressor. No difference in terms of norepinephrine hours of administration was reported between dysoxic and vasoplegic shock cases.

Three cats initially diagnosed with cryptic shock developed dysoxic shock within 24 h.

### Comparison Between Cats With Septic Shock vs. Cats With Uncomplicated Sepsis

The comparison of selected clinical and clinicopathological variables between cases with uncomplicated sepsis and septic shock is summarized in Table 3. Cats with septic shock had a significantly higher frequency of MODS (91% vs. 28%, *P* < 0.0001) and a significantly higher mortality rate (73% vs. 43%, *P* = 0.0085) than cats with uncomplicated sepsis.

In the overall population of enrolled cats, patients presenting with at least 2/4 SIRS criteria had significantly higher frequency of MODS (76% *vs*. 24%, *P* = 0.022) and mortality (67% *vs*. 36%, *P* = 0.01) compared to those not fulfilling the feline SIRS criteria.

### Comparison Between Cryptic Shock vs. Uncomplicated Sepsis

Additional comparisons were made between cryptic shock cases and cats with uncomplicated sepsis. No significant differences were identified for APPLE_fast_ score (27 *vs*. 27, *P* = 0.22), MODS occurrence (57% *vs*. 29%, *P* = 0.30) and mortality rate (57% *vs*. 43%, *P* = 0.68).

### Comparison Between Survivors vs. Non-survivors

In the overall population of enrolled cats, 48/85 (56%) died, of whom 26/48 (54%) were euthanized. Selected clinical and clinicopathological variables in survivors and non-survivors are reported in Table 4. Non-survivors had significantly lower body temperature and BCS, as well as lower hematocrit, lymphocyte count and serum albumin compared to survivors. Moreover, circulating methemoglobin, serum bilirubin, serum urea, activated partial thromboplastin time and APPLE_fast_ score were higher in non-survivors. Non-survivors had greater occurrence of MODS compared to survivors (74% *vs*. 38%, *P* = 0.0033), and presented overall a shorter duration of hospital stay. Variables that were significantly different between survivors and non-survivors were entered into a univariate logistic regression analysis. There were positive associations between odds of mortality and body temperature, lymphocyte count, APPLE_fast_ score, low BCS (1–3) and MODS occurrence. MODS and lymphocyte count were the only variables retained in the multivariate model (OR = 4.3, CI 1.5–12.4; OR = 0.99, CI 0.99–1.0, respectively).

**Table 4:** Clinical and clinicopathological variables in survivors and non-survivors in the study population.

**Variable**	**RI**	**Survivors (*n =* 37)**	**Non-survivors (*n =* 48)**	***P*-value**
**Clinical data**				
Weight (kg)		4.1 (1.0–8.0)	3.4 (0.5–9.4)	**0.0055**
Body temperature (°C)	38.0–39.0	38.6 (32.0–40.5)	37.0 (32.0–40.6)	**0.0005**
Heart rate (bpm)	160–220	184 ± 35	171 ± 38	0.1124
Respiratory rate (rpm)	10–40	32 (20–100)	28 (20–100)	0.3150
SBP (mmHg)	120–170	108 (70–167)	95 (51–185)	**0.0066**
MAP (mmHg)	60–130	88 ± 25	74 ± 26	**0.0143**
BCS		3.9 ± 1.4	3.1 ± 1.9	**0.0043**
APPLE fast score		27.6 ± 8.9	32.1 ± 8.3	**0.0181**
APPLE full score		49.5 ± 13.2	46.5 ± 10.7	0.4144
SIRS citeria (*n*)		1.8 ± 1.3	2.2 ± 0.9	**0.0383**
Length of hospital stay		7 (2–28)	2 (0–12)	** <0.0001**
**Blood gas analysis**				
pH	7.31–7.46	7.29 (6.92–7.40)	7.25 (6.91–7.44)	0.3746
pC*O*_2_ (mmol/L)	32.7–44.7	35.1 (22.2–58.8)	37.3 (7.6–66.4)	0.6369
HC*O*_3_ (mmol/L)	18.0–22.0	17.0 (4.5–30.7)	16.6 (3.0–26.8)	0.6248
BE (mmol/L)	−2.0–2.0	−8 (-29–6.2)	−9.8 (-24.2–2.6)	0.4096
Ionized calcium (mmol/L)	1.10–1.40	1.21 (0.96–1.36)	1.22 (0.72–1.66)	0.6695
MetHb (%)	0.0–2.1	1.3 (0.0–4.3)	1.7 (0.8–6.4)	**0.0415**
Lactate (mmol/L)	0.5–2.0	2.2 (0.9–26.0)	3.2 (0.5–14.4)	0.3218
**Hematology**				
HCT (%)	24.0–45.0	31.3 ± 7.8	27.6 ± 7.9	**0.0328**
Hb (g/dL)	10.6–15.6	10.5 ± 2.9	9.1 ± 2.8	**0.0284**
WBC (× 10^9^/L)	5.0–19.0	12.7 (0.6–66.1)	10.2 (0.0–96.6)	0.3487
Lymphocytes (× 10^9^/L)	1.5–7.0	1.2 (0.2–3.2)	0.7 (0.0–2.9)	**0.0119**
Platelets (× 10^9^/L)	300.0–700.0	239.4 ± 113.3	193.2 ± 134.3	0.0976
**Chemistry**				
Glucose (mg/dL)	75–160	144 (49–367)	135 (11–702)	0.0789
Creatinine (mg/dL)	0.80–1.80	1.31 (0.46–23.04)	1.29 (0.26–19.47)	0.8835
Urea (mg/dL)	15–60	53 (24–797)	119 (16–868)	**0.0111**
Total proteins (mg/dL)	6.0–8.0	6.4 (4.7–10.3)	5.9 (3.7–10.3)	**0.0241**
Albumin (mg/dL)	2.1–3.3	2.7 ± 0.6	2.4 ± 0.7	**0.0412**
CK (U/L)	0–120	510 (62–570,000)	519 (87–249,000)	0.7892
Cholesterol (mg/dL)	96–248	145 (50–310)	212 (11–702)	**0.0024**
Total bilirubin (mg/dL)	0.0–0.70	0.27 (0.10–7.46)	0.70 (0.09–9.19)	**0.0120**
Magnesium (mg/dL)	1.9–2.6	2.5 (1.3–7.2)	3.3 (1.2–6.7)	**0.0432**
SAA (mg/dL)	0–10	125 (1–316)	174 (1–338)	0.6028
**Coagulation**				
PT (sec)	0.0–15.0	9.6 (7.4–18.9)	10 (5.4–20.3)	0.9506
aPTT (sec)	0.0–20.0	17.6 (8.7–45.9)	32.4 (11.2–180.0)	**0.0033**

*Data are reported as mean±standard deviation or median and range (minimum-maximum value), based on their distribution*.

*APPLE, acute patient physiologic and laboratory evaluation; aPTT, activated partial thromboplastin time; BCS, body condition score; BE, base excess; CK, creatine kinase; HCT, hematocrit value; MetHb, methemoglobin; PT, prothrombin time; RI, reference interval; SAA, serum-amyloid A; SIRS, systemic inflammatory response syndrome; WBC, white blood cell. Bold indicates significance*.

## Discussion

This prospective study includes a large population of cats with sepsis and septic shock, and explores the features of three septic shock phenotypes based on the presence of hypotension, hyperlactatemia, or both. In this regard, we hypothesized that the prognostic implications of the three septic shock phenotypes in cats resemble the ones reported in humans.

Demographic data, sepsis etiologies and mortality rates in our study population were comparable with the results of previous studies in septic cats (2, 3, 15, 17, 19, 20). Moreover, we were able to identify all three septic shock phenotypes with the dysoxic shock being the most represented. However, differences in terms of major outcomes emerged in contrast to human literature. Length of hospital stay was not different among the septic shock phenotypes. Mortality rates increased from cryptic (57%) to dysoxic (65%) and vasoplegic (91%) shock, but the difference was not significant. Moreover, no difference was detected in terms of vasopressor requirements (hours of administration) between dysoxic and vasoplegic shock phenotypes. Both dysoxic and vasoplegic shock were characterized by an overall greater illness severity, with higher numbers of patients with more frequent occurrence of MODS and higher APPLE scores. Hence, in feline sepsis, fluid-refractory hypotension seems to carry an elevated risk of morbidity and organ dysfunction regardless the presence of concurrent hyperlactatemia. Thus, even if a mortality difference was not apparent, the different morbidity of the three septic shock phenotypes could be equally relevant in the context of veterinary medicine, where the possibility of euthanasia confounds disease course and outcome in a notable way.

Unexpectedly, cats with cryptic shock were indeed comparable in terms of illness severity, outcome and MODS frequency to the ones affected by uncomplicated sepsis. In addition, lactate concentration upon hospital admission was similar between survivors and non-survivors. The prognostic utility of lactate concentrations in cats appears unclear according to the available literature. In the study by Shea et al. (21) investigating blood lactate in cats with arterial hypotension, normolactatemic patients had higher blood pressure and higher survival rates compared to hyperlactatemic patients. The authors hypothesized that normolactatemia in hypotensive patients could indicate an early state of disease with preserved organ perfusion. In a study including sick cats evaluated at an emergency service, patients with abnormal perfusion variables (pale mucous membranes, hypothermia, poor pulse quality) had higher lactate concentrations compared to cats without these findings, possibly indicating severe shock and altered tissue oxygen delivery (21). Nonetheless, neither initial or serial lactate measurements have shown prognostic significance in sick cats (22, 23). Despite the similar findings, comparison between these and our results is difficult considering the differences in patient cohorts and specifically their illness severity. It might be possible that blood lactate has a weaker prognostic value in septic cats compared to humans and dogs (24), but the impact of potential confounders (e.g., lack of standard sampling site, impact of restraint, type B hyperlactatemia, low number of cases per group) has to be considered.

Besides hypotension and hyperlactatemia, cats with septic shock had significantly lower body temperature compared with the ones affected by uncomplicated sepsis. Hypothermia is commonly included in the triad of feline shock, but its occurrence linked with severe sepsis and septic shock has been documented only in few of studies (15, 20). Hence, hypothermia even during triage should immediately prompt complete patient screening inclusive of blood pressure and lactate measurement in order to better assess perfusion. Cats with septic shock had higher APPLE_fast_ score, MODS occurrence and mortality rate compared to cats with uncomplicated sepsis. These results, overall, corroborate the concept of septic shock as the subset of sepsis characterized by substantially greater severity and worse prognosis.

The mortality rate in the overall study population was 56%. Non-survivors were generally characterized by more severe clinical and clinicopathological abnormalities, including lower body temperature and higher APPLE_fast_ score compared to survivors.

Interestingly, most of the cats in our population presented with a low-normal BCS, and being underweight was associated with an increased risk of death. It has been previously showed that a poor nutritional condition has a negative impact on wound repair, immune function, strength of skeletal and respiratory muscles, and might negatively affect outcome in hospitalized cats (25). Our results might support this statement, although a cause-effect relationship cannot be demonstrated. Among hematological variables, non-survivors had significantly lower lymphocyte count compared to survivors. Lymphocyte count was significantly associated with death in both univariate and multivariate analyses. Lymphopenia in cats usually accompany an inflammatory leukogram, but might also suggest a state of ineffective immune response. However, to the author's knowledge, it has not previously emerged as an outcome predictor in feline sepsis. Interestingly, apoptosis-induced lymphopenia is documented in septic people, and both its duration and depth have been linked with poor prognosis (26).

In the current study, MODS occurrence had a major impact on final outcome, being associated with a 5-fold increase in the risk of death in the multivariate regression analysis. Sepsis-associated mortality is highly related to the development of MODS in people (27). As a syndrome, MODS is intimately related to the individual host response to infection, and recognizes several mechanisms including disruption of microcirculation, endothelial damage, mitochondrial dysfunction and uncontrolled apoptosis (27). Previous studies have shown that MODS is a frequent complication in septic cats and similarly to this study has been associated with worse outcomes (3). Thus, our results strongly support frequent organ function assessment in septic cats, since early MODS detection can be crucial in preventing its progression.

Based on the criteria applied for the present study, 28/85 (33%) cats were considered septic despite showing <2/4 SIRS criteria. This result demands consideration, as sepsis could have been missed in about one third of the overall study population if only the traditional criteria proposed for cats were used (2). Despite the low sensitivity and specificity for sepsis diagnosis, the SIRS criteria might still have a place in identifying cats with higher disease severity. Indeed, according to our results, meeting the SIRS criteria was associated with worse outcomes and more frequent occurrence of MODS. On the contrary, SAA concentration was comparable between cats with uncomplicated sepsis and septic shock, as well as between survivors and non-survivors. Similar results had been previously reported, and indicate that despite SAA having a great value to recognize systemic inflammation in cats, its prognostic utility seems poor (15, 17).

There are some limitations to acknowledge when interpreting our results. The present study defined sepsis as the combination of infection plus either fulfillment of two or more SIRS criteria, increased SAA concentrations or evidence of new-onset organ dysfunction. These are novel criteria for sepsis in the veterinary literature. Our choice was based on multiple reasons. The SIRS criteria were insensitive to cats with confirmed infection and organ dysfunction in the study by Babyak and Sharp (2), and SAA has been demonstrated to be an excellent marker of systemic inflammation in cats (17). In human medicine, a data driven approach has been conducted to derive novel clinical criteria for sepsis redefinition and identification (1). An equivalent redefinition of sepsis in veterinary medicine has not been undertaken; however, in the Authors opinion, the assessment of acute phase proteins and markers of organ function to supplement SIRS criteria could be a strategy to apply to ameliorate sepsis identification. Similarly, the present study defined septic shock based on the presence of persistent hyperlactatemia or the need of vasopressors to support blood pressure. While hyperlactatemia has been recently incorporated into the human sepsis 3 definitions (1), no clear role for persistent hyperlactatemia has been defined in veterinary medicine. Another limitation of our study was intrinsically related to blood pressure measurement performed using a non-invasive indirect technique, which could be inaccurate especially in the hypotensive setting. Moreover, this was a single-center study; hence, our results might not be extrapolated in different settings. No sample size calculation was performed and, despite the reasonable size of the whole cohort of septic and septic shock cats, the number of cases per-group was small. According to the mortality rates detected in the present study a minimum number of 25 patients per group would have been necessary to detect a difference between vasoplegic and cryptic shock cases (type I error 0.05 and 80% power). This preliminary evidence limits the statistical power of our comparisons. Diagnostic and therapeutic interventions were left at the discretion of the attending clinician, thus no standardization in terms of blood sampling site was performed and peripheral and jugular lactate measurements were used interchangeably. Nonetheless, a pilot study did not report a significant difference between cephalic and jugular lactate in cats, even in the presence of hypotension (28). This shows that the sampling site might have limited impact on obtained lactate concentrations in cats. Cats were considered affected by cryptic shock only after the presence of sustained hyperlactatemia despite euvolemia and adequate fluid resuscitation, but the timing for lactate monitoring was not standardized, and no struggling score was calculated. However, the majority of cats with cryptic shock remained hyperlactatemic for a period of time ranging from 12 to 24 h, corroborating the possibility of an actual sustained hyperlactatemia refractory to fluid therapy. We did not attempt to differentiate between type A and type B hyperlactatemia. Specifically, among the factors and comorbidities potentially linked with type B hyperlactatemia there were steroid therapy (*n* = 3), diabetic ketoacidosis (*n* = 2) and hepatic lipidosis (*n* = 1). Those patients, and others with unknown predisposing factors, might have had hyperlactatemia without any metabolic, cellular or perfusion derangement. Interestingly hyperlactatemia B does not seem to be distinguished from hyperlactatemia A in studies in people with cryptic shock (4–6). According to our study design, the APPLE_fast_ and APPLE_full_ scores were calculated for cats in septic shock, while only the APPLE_fast_ score was evaluated in cats with uncomplicated sepsis for financial and case management reasons. This choice prevented a further comparison between these groups. In addition, the lack of external validation concerning the APPLE scores prevents us to get any strong conclusion regarding their clinical relevance in our population. Finally, despite exclusion of cats with financially-driven euthanasia, the number of cats euthanized for disease severity was high, limiting the value of the comparisons concerning length of hospital stay and outcome.

## Conclusion

Septic shock in cats defines a subset of patients with greater disease severity, organ dysfunction and mortality compared to uncomplicated sepsis. At least three different septic shock phenotypes can be identified in feline sepsis. Despite similar in-hospital mortality, cats with dysoxic and vasoplegic shock are characterized by greater organ dysfunction compared to cats affected by cryptic shock. When compared to cats affected by uncomplicated sepsis, cryptic shock cases were not different in terms of illness severity and MODS occurrence. This new spectrum of feline septic shock phenotypes based on hypotension and hyperlactatemia warrants further research in larger study populations to implement epidemiological data and better delineate prognostic implications.

## Data Availability Statement

The raw data supporting the conclusions of this article will be made available by the authors, without undue reservation.

## Ethics Statement

The project was approved by the Animal Welfare Committee (COBA) of the Alma Mater Studiorum – University of Bologna (Bologna ID 846).

## Author Contributions

RT and MG designed the study, analyzed the data, co-wrote, and edited the manuscript. FB, EC, and AF analyzed the data, co-wrote, and edited the manuscript. IM, GM, and FD edited the manuscript. All authors contributed to read and approved the final manuscript.

## Conflict of Interest

The authors declare that the research was conducted in the absence of any commercial or financial relationships that could be construed as a potential conflict of interest.

## Publisher's Note

All claims expressed in this article are solely those of the authors and do not necessarily represent those of their affiliated organizations, or those of the publisher, the editors and the reviewers. Any product that may be evaluated in this article, or claim that may be made by its manufacturer, is not guaranteed or endorsed by the publisher.

## Abbreviations

APPLE_fast_: Acute Patient Physiologic and Laboratory Evaluation fast score
APPLE_full_: Acute Patient Physiologic and Laboratory Evaluation full score
BCS: body condition score
CI: confidence interval
%FO: percentage of fluid overload
ICU: intensive care unit
OR: odds ratio
MAP: mean blood pressure
MODS: multiorgan dysfunction syndrome
SBP: systolic blood pressure
SIRS: Systemic Inflammatory Response Syndrome
SAA: serum amyloid A.
